# Degradation Mechanisms of Metal-Supported Solid Oxide Cells and Countermeasures: A Review

**DOI:** 10.3390/ma14113139

**Published:** 2021-06-07

**Authors:** Zhipeng Zhou, Venkata Karthik Nadimpalli, David Bue Pedersen, Vincenzo Esposito

**Affiliations:** 1 Department of Energy Conversion and Storage, Technical University of Denmark, 2800 Kgs Lyngby, Denmark; zhizho@dtu.dk; 2 Department of Mechanical Engineering, Technical University of Denmark, 2800 Kgs Lyngby, Denmark; vkna@mek.dtu.dk (V.K.N.); dbpe@mek.dtu.dk (D.B.P.)

**Keywords:** metal-supported oxide cells, degradation mechanisms, countermeasures

## Abstract

Metal-supported oxide cells (MSCs) are considered as the third-generation solid oxide cells (SOCs) succeeding electrolyte-supported (first generation) and anode-supported (second generation) cells, which have gained much attention and progress in the past decade. The use of metal supports and advanced technical methods (such as infiltrated electrodes) has vastly improved cell performance, especially with its rapid startup ability and power density, showing a significant decrease in raw materials cost. However, new degradation mechanisms appeared, limiting the further improvement of the performance and lifetime. This review encapsulates the degradation mechanisms and countermeasures in the field of MSCs, reviewing the challenges and recommendations for future development.

## 1. Introduction

**Figure 1:**
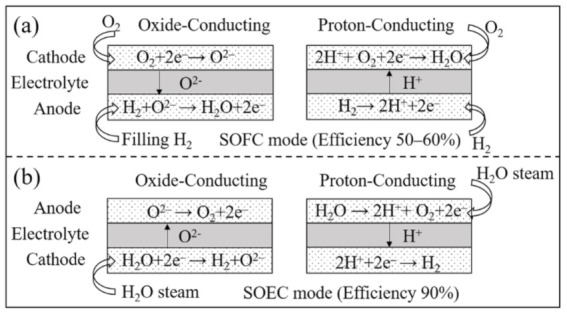
Schematic diagram of the structure and operating principle of SOFCs and SOECs. (**a**) SOFC mode with an oxide-conducting and proton-conducting electrolyte, respectively; (**b**) SOEC mode. An energy conversion efficiency of 50–60% can be achieved in SOFC mode [1] and 90% in SOEC mode [8].

Solid oxide cells (SOCs) are electrochemical devices, including functional metal oxides operating either as fuel cells or electrolysers at high temperatures. Solid oxide fuel cells (SOFCs) are energy conversion devices that convert a fuel’s chemical energy into electricity through a series of electrochemical reactions. A typical structure and the operating principle of SOFCs are shown in Figure 1a. The three main components are a dense electrolyte, a porous anode, and a porous cathode, which make up a single SOFC. According to the type of conductor, it can be divided into oxide-conducting and proton-conducting SOFCs. The electrical efficiency can typically reach 60% when hydrogen is used as fuel [1]. Such a performance is higher compared to other commercially viable fuel cells such as phosphoric acid fuel cells (PAFC; 40–45%) and proton exchange membrane fuel cells-(PEMFC; 40–50%) [2]. Among several advantages, the all-solid structure of SOFCs prevents the risk of electrolyte leakage. The heat produced during the operation can be reused, increasing the total efficiency to more than 80%. SOFCs are commended further, from that, relatively high operating temperatures compared with PEMFC allows inexpensive metals such as nickel and copper to become catalysts, meanwhile, the high ionic conductivity of the electrolyte can be achieved at these temperatures [3,4,5,6]. Furthermore, it can be changed from SOFC to SOEC (solid oxide electrolysis cell) mode with almost no adjustment other than the water steam be filled in. The operating process of SOEC is shown in Figure 1b. Higher efficiencies around 90% can be achieved in SOEC mode compared to conventional low-temperature electrolysers (75%) [7,8].

**Figure 2:**
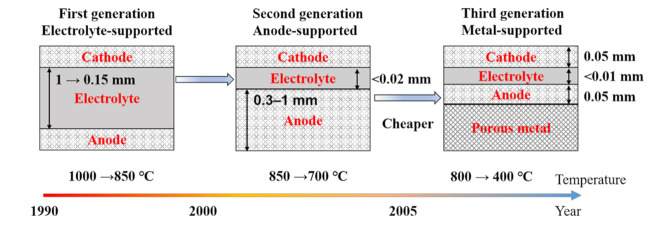
Schematic diagram of the development of SOFCs [9,22]. Note the thinner electrolyte allows a lower operating temperature, and the low operating temperature allows the use of metal supports.

These advantages have given SOFCs a great deal of attention in the past few decades. In the early stages, the electrolyte-supported mode was primarily used for SOFCs with the structure shown in Figure 2. The dense yttria-stabilized zirconia (YSZ) of more than 0.15 mm in thickness was used as an electrolyte to support the anode and cathode [9]. A thick electrolyte needs a high operating temperature to reduce its ohmic impedance during the operation because the ohmic impedance is proportional to the thickness while displays Arrhenius dependence on temperature [10]. Thus, for an electrolyte-supported cell (ESC), the operating temperature was usually set at about 1000 °C in the early years.

The high operating temperature increases electrodes’ coarsening rate and the risk of reaction between the cathode and electrolyte, leading to a decrease in the performance and lifetime of ESCs [5,11]. The high operating temperature also poses a challenge to sealing and matching the interconnects with electrodes, increasing the cost [12,13]. Therefore, an intermediate operating temperature has been an aim for the development of economically viable SOFCs. With the development of ceramic processing techniques such as tape casting, tape calendaring, slurry sintering, and screen printing, the thickness of the electrolyte can be reduced to 20 μm and even less than 10 μm. The operating temperature can be reduced to 800 °C and even lower [9,14,15,16,17,18]. With the application of advanced technologies such as pulsed laser deposition (PLD), sputtering, and suspension plasma spraying (SPS), the thickness of the electrolyte can be further reduced to less than 1 μm, which allows the operating temperature down to 400 °C [17,19,20,21,22]. The decreasing operating temperature allows for metal supports (such as nickel and ferritic stainless steel) with significant advantages. Firstly, the application of inexpensive metal supports can reduce the cost of SOFCs, because costly anode can be released from the role of mechanical support to allow a five-times decrease at least in thickness, as shown in Figure 2. The raw material cost decreases from 60–96 USD/kg to 12–24 USD/kg when the anode support is replaced by metal support [9]. Moreover, metal-supported cells (MSCs) show much better stability in the rapid thermal and redox cycling than ASCs as the metals’ excellent ductility and high thermal conductivity, which can significantly mitigate the thermally induced stress during the operation [6,23,24,25,26]. These excellent physical properties allow the cell stack with metal-supported SOFCs more compact to get higher power density and also to be with superior fast-startup capability [27,28,29,30,31].

**Figure 3:**
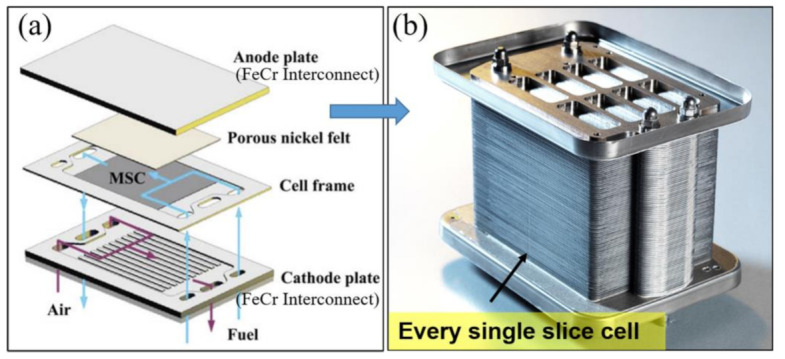
(**a**) Diagram of MSCs stack’s structure, reproduced with permission from Ref. [35]; (**b**) 1 kW MSCs stack manufactured by Ceres Power, reproduced with permission from Ref. [36].

The individual cells are integrated into a stack through connectors to provide sufficient power for the applications. A typical planar type MSCs stack is shown in Figure 3. As a benefit from the high power density and fast startup capability of MSCs, the cell stacks own the better applicability for auxiliary power units (APUs) and other mobile applications compared with conventional ceramic-supported cell stacks. They have been developed for direct placement in charcoal cooking stoves [32,33], home-scale combined heat and power [34], and propane-fueled personal device chargers [16].

MSCs show a promising application prospect, yet there are still open challenges. On the one hand, the degradation that occurred in ASCs also occurred in MSCs, such as nickel coarsening [23,37,38,39,40], carbon deposition [41,42,43,44,45,46], sulfur poisoning [47,48,49,50,51], and chromium poisoning [5,52,53]. On the other hand, introducing metals in the SOC system also presented some additional degradation mechanisms, such as the oxidation of the metal support [54,55,56] and metal elements diffusion between the electrode and metal support [5,52,57]. These are the main factors to restrict the performance and lifetime of MSCs, which have received significant attention from researchers in recent years. This paper aims to provide an overview of the main degradation mechanisms and current countermeasures of MSCs, to provide a basis for further development.

## 2. Degradation Mechanism and Countermeasures

### 2.1. The Degradation of the Anode

The anode of MSCs can be divided into the functional layer and supporting layer. The porous functional layer provides triple-phase boundaries (TPB), i.e., the collection of the sites where the electron- (such as nickel), ion- (such as YSZ), and gas (pore)-conducting phases meet for the electrochemical reaction. Phase connectivity is required to maintain the gas diffusion and the conduction of electrons and ions, keeping TPBs active and cells working. For the supporting layer, providing mechanical support, conducting gas, and electricity are its main functions. Therefore, all the changes that make TPBs and the metal support ineffective constitute the anode’s degradation mechanisms.

#### 2.1.1. Coarsening and Migration of Nickel

**Figure 4:**
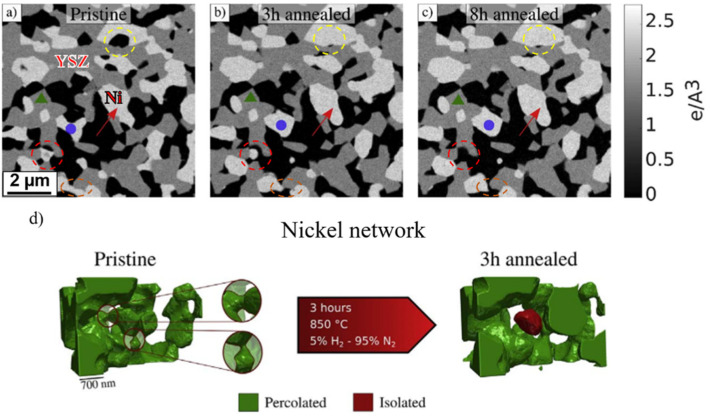
Two-dimensional slices from a spatially registered sub-dataset at identical locations in the electrode in the pristine (**a**), annealed for 3 h (**b**) and 8 h (**c**) states. Three different grey levels are present: black (pore), grey (YSZ), and white (nickel). (**d**) The nickel particle morphology at the same position before and after the annealing (in dry hydrogen at 850 °C). Reproduced with permission from Ref. [40].

Anodes consisting of nickel and yttria-stabilized zirconia (Ni/YSZ) (ionic conductor) and gadolinium-doped ceria (Ni/GDC) (mixed conductor of ions and electrons) are the most widely used because Ni has good catalytic activity for the oxidation of hydrogen [9]. The coarsening of nickel is a common degradation mechanism for both MSCs and ceramic-supported SOCs. Tucker et al. (2007) [37] used NiO as the precursor to be co-sintered with YSZ and the metal support in reducing atmosphere (4% H_2_/96% Ar) at 1300 °C for 4 h, which was a conventional process to fabricate the porous anode [9,58,59]. However, the particle size increased from 3 μm to 10 μm when NiO was reduced to nickel. Nickel coarsening is primarily caused by Ni-Ni inter-diffusion [38,39,40]. The mass transfer rate is thermally activated, and the flow is from the high chemical potential point to the low chemical potential point [38,40]. Such directional diffusion causes a progressive coarsening of nickel particles, leading to isolated particles’ appearance and TPBs’ length decrease. Angelis et al. (2018) [40] demonstrated this coarsening process in three dimensions via the ex situ ptychographic nanotomography method, as shown in Figure 4. Ni-YSZ anode was annealed at 850 °C in a mixture of hydrogen (5%) and nitrogen (95%). Fast diffusion occurred at the beginning of annealing, resulting in isolated nickel particles within 3 h.

**Figure 5:**
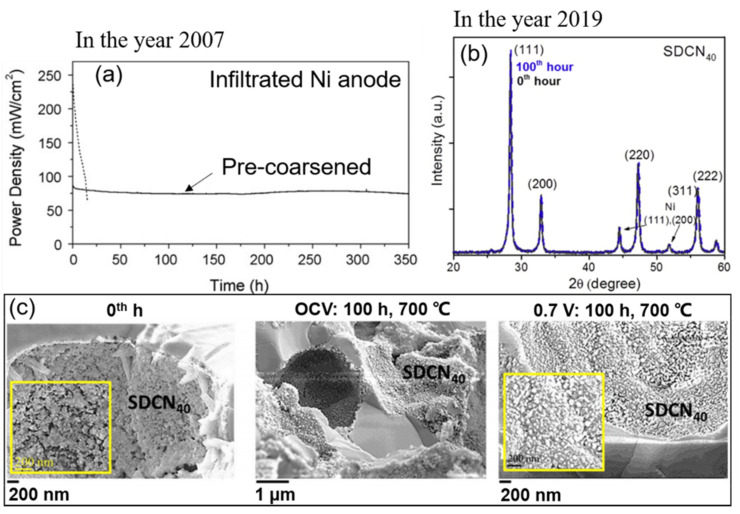
(**a**) Operation of tubular metal-supported SOFCs at 700 °C with moist hydrogen fuel and oxygen as oxidant. Dashed line: as-infiltrated Ni anode, 300 m Acm^−2^; solid line: Ni anode was pre-coarsened at 800 °C, 100 mA cm^−2^, reproduced with permission from Ref. [23]. (**b**) X-ray diffraction patterns for SDCN_40_ anode catalyst upon reduction at 700 °C for 1 h (black) and after 100 h of thermal annealing at 700 °C in 3% humidified hydrogen (blue). (**c**) SDCN40 catalyst anode via thermal annealing at 700 °C and continuous electrochemical operation at 0.7 V for 100 h (corresponding to (**b**)). (**b**,**c**) are reproduced with permission from Ref. [52].

The most common countermeasure for Ni-coarsening is nickel nanostructuring. Mainly, infiltrated anodes have been used to avoid nickel coarsening caused by the co-sintering [23,28,60]. Nickel nanoparticles were infiltrated into the porous backbone of YSZ. This method prevents the nickel from coarsening at high temperatures during the co-sintering process. Moreover, infiltrating the anode with nanoparticles can mitigate the degradation caused by nickel migration. Ovtar et al. (2019) [61] infiltrated Ce_0.8_Gd_0.2_O_2−δ_ (CGO) nanoparticles into the Ni/YSZ electrode. They tested the cell at 800 °C in SOEC mode, and even after a test of 1000 h, no isolated nickel particles were observed. The strong adhesion of CGO (GDC) nanoparticles on the nickel surface and the heterogeneous mass diffusion mechanisms in nanocomposites may impede surface diffusion [62,63]. Although infiltrated electrodes show significant advantages over traditional electrodes, the coarsening and aggregation of the nano-nickel often appear, caused by the high diffusive activity of the nanoparticles [23,52]. Early work by Tucker et al. (2008) [23] showed infiltrated nano-nickel particles of the anode coarsened during the operation (700 °C), resulting in rapid degradation of the MSC, as shown in Figure 5a (dashed line). Thermochemical treatments were thus adopted as a countermeasure to keep a stable microstructure. The nanoparticles were pre-coarsened at a higher annealing temperature (800 °C) than the operating temperature (700 °C) in a mixture of hydrogen (4%) and argon (96%). As a result, the cell had a stable operation but a significant performance loss (Figure 5a). Blennow et al. (2009) [64] demonstrated that mixed nano-sized CGO (GDC) with nano-nickel to form the conductive cermet anode (Ce_0.8_Gd_0.2_O_2−δ_ + 10 wt.% Ni) could stabilize the nickel particles. Such small amounts of nickel were sufficient for catalysis and would be beneficial to reduce nickel agglomeration risk. Moreover, a subsequent work (2011) confirmed that the MS-SOFC with this novel anode (Ce_0.8_Gd_0.2_O_2−δ_ + 10 wt.% Ni) could achieve a power density of around 500 mW/cm^2^ at 650 °C (fuel: moist hydrogen; oxidant: air), with a degradation rate of 4.5%/1000 h [65]. Thus, the nickel coarsening for an infiltrated anode seems can be avoided at present. E. Dogdibegovic et al. (2019) [52] demonstrated that the infiltrated SDCN_40_ (Sm_0.20_Ce_0.80_O_2−δ_ + 40 wt.% Ni) anode of MSC underwent almost no coarsening after the 100-h annealing at 700 °C in 3% humidified hydrogen, as shown in Figure 5b,c.

As well as infiltrated electrodes, the in situ exsolution method is another way to get nano-structure electrodes, which has also attracted a lot of attention in recent years [66,67,68,69,70]. Tan et al. [67] (2018) achieved uniform-distributed nickel nanocatalyst on GDC surface using a thermally driven in situ exsolution method in the SOC system. The isolated Ni nanoparticles on the surface of GDC can effectively increase TPB density while avoiding the agglomeration of nanoparticles which is usually caused by the infiltration method. A power density of around 1 W/cm^2^ can be achieved at 650 °C in humid H_2_. However, the isolated Ni particles decreased the electron conductivity of the system, leading to an increase in ohmic resistance. Moreover, an annealing process of 1250 °C in the air is required to recover the GDC phase from GNDC [67], limiting the application of the method in MSCs.

#### 2.1.2. Metal Support Issues

Ni [71,72,73,74], Ni-Fe alloys [75,76,77,78,79], and ferritic stainless steels [57,80,81,82,83] have been mostly used as metal supports from 2000 to 2020. Table 1 shows a comparison between Ni, Ni-Fe, and 400-series stainless steels (ferritic stainless steels) in the coefficient of thermal expansion (CTE) and relative oxidation resistance [5]. Ferritic stainless steels (FSS) have the best oxidation resistance among them (Table 1). The dense chromium oxide film on the FSS surface can effectively alleviate the oxidation rate even at high temperatures [9]. Oxide scales (NiO and Fe_2_O_3_) also can be formed on the surface of nickel and iron, but their porosity is higher than Cr_2_O_3_, especially for Fe_2_O_3_, leading to the worse oxidation resistance of Ni or Ni-Fe than that of FSS. Although pure nickel has a low antioxidant ability and large CTE, it had been often used as metal supports, especially before 2010. Firstly, nickel support can meet the dual needs of catalysis and mechanical support. Furthermore, the element diffusion between the anodic functional and support layers can be avoided. The diffusion of Fe and Cr in the support layer into the nickel in the functional layer leads to the failure of the catalyst caused by the formation of insulating oxides such as Cr_2_O_3_, NiCr_2_O_4_, and Fe_2_O_3_. The diffusion of Ni into the support leads to the austenitic transformation to increase the CTE and reduce the oxidation resistance. Thus, a significant degradation often appeared when stainless steels were used as support in the early years [5,37,73]. However, the large CTE of pure nickel posed a challenge for co-sintering with the ceramic electrolyte. Thus, Ni-Fe alloy was regarded as an alternative because of its smaller CTE [75,76,77,78,79]. The low oxidation resistance of Ni-Fe limits the long-term stable operation of the Ni-Fe support cells. Ni-Fe alloy does not contain chromium, cannot form the oxide scale (Cr_2_O_3_) on the surface to prevent further oxidation, and will be rapidly oxidized when exposed to a high-temperature and humid atmosphere. The set of failures together causes a gradual loss in the conductivity, limiting the lifetime of MSCs significantly. Thus, pure nickel, Ni-Fe alloys, and ferritic stainless steels all showed insurmountable limitations for the use of metal support in the early years.

**Figure 6:**
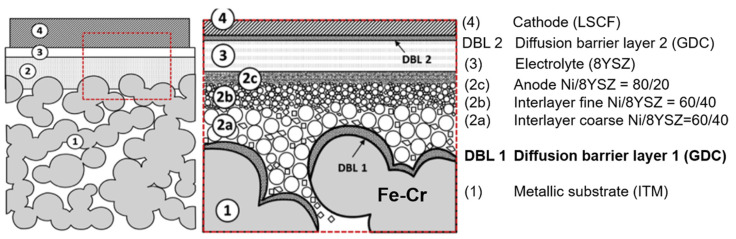
Schematic of the diffusion barrier layer method in metal-supported cells, reproduced with permission from Ref. [57].

In recent years, the application of the diffusion barrier layer (DBL) method has relieved the issues caused by elements diffusion between the anode and metal support, which allows the unrestricted use of ferritic stainless steel for the support [84]. Thereupon, ferritic stainless steels have gained prominence due to their low cost and good CTE matching. The ‘DBL-1′ prevents element diffusion between the Ni-containing anode and Fe-Cr support, and ‘DBL-2′ mitigates the cathode’s degradation [85,86,87] (Figure 6). Mixed ions and electrons conductor GDC with a low CTE value of 12.7 ppm/K is used for DBLs mostly [57]. The DBL with a thickness of 1–2 µm can be deposited by a series of techniques such as atomic layer deposition (ALD) [52,57], atmospheric plasma spray (APS) [72,88], pulsed laser deposition (PLD) [9], and physical vapour deposition (PVD) [84,89]. The element diffusion rate can be reduced from 17%/200 h to 0.1%/200 h [90] with a DBL. Therefore, the DBL method seems to make ferritic stainless steel to be the most promising metal material for support.

**Figure 7:**
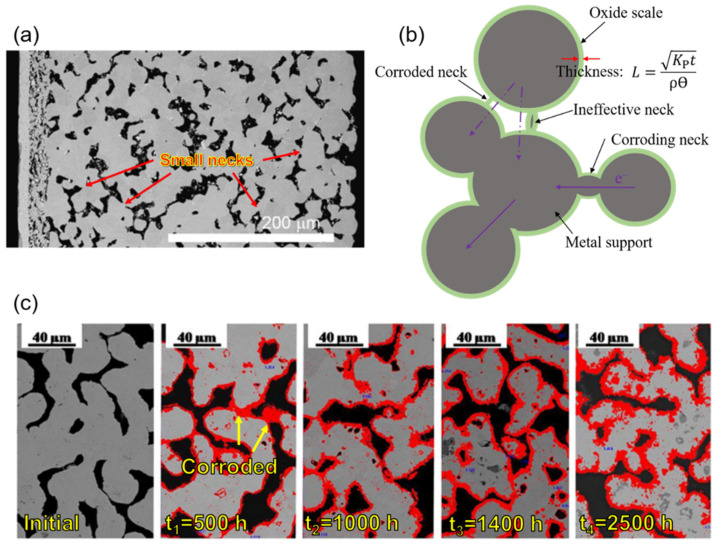
(**a**) SEM image showing the polished cross-section of the planar metal-supported half-cell. The electrolyte is shown at the top, followed by the cermet layer and the metal support, reproduced with permission from Ref. [65]. (**b**) Schematic diagram of the loss of conductivity of the metal support due to oxidation adapted from Ref. [9]. (**c**) Cross-sectional SEM images of the metal support with a grown outer oxide layer in darker grey at different exposure times at 800 °C and 50%H_2_-50%H_2_O fuel atmospheres, reproduced with permission from Ref. [90].

Although ferritic stainless steels have excellent oxidation resistance because of their unique chemical compositions, the lifetime of metal supports also strongly depends on the structure and operating temperature. Conventional powder metallurgy methods, such as tape casting, are primarily used to fabricate porous metal supports. The random-distributed pores with irregular shapes and small sintering necks are pervasive in the metal support, as shown in Figure 7a.

The dense Cr-oxide scale will distribute on the metal support surface to suppress further oxidation. However, the oxide scale will grow with time (Figure 7b), the thickness *L* can be presented by the equation [9]:

(1) $$L=\frac{\sqrt{K_{p}t}}{\rho \theta }$$

where *L* is the oxide scale thickness (cm), *t* is time (s), *ρ* is the density of the scale (g cm^−3^), and *θ* is the weight fraction of oxygen in the scale. *K*_P_ is the parabolic growth rate constant, which is usually used to evaluate the corrosion rate (oxidation resistance). For standard dense metal samples, *K*_P_ can be described as the following equation [91]:

(2) $$K_{P}=\frac{\Delta m^{2}}{A^{2}t}$$

where Δ*m* is the weight gain of the sample (g), *A* is the sample surface area (cm^2^), and *t* is time (s). Here, the unit of *K*_P_ is g^2^ cm^−4^ s^−1^. As for porous metal samples, the equation is usually modified as [92]:

(3) $$K_{P}\left({\%}^{2}\right)=\frac{\Delta m^{2}}{m^{2}t}$$

where m is the original weight of the sample (g), Δ*m* is the weight gain of the sample (g), and *t* is time (s). Here, the unit of *K*_P_ can be presented as %^2^/s. The thickness of the oxide scale increases typically with time according to Equation (1), and the conductivity of a narrow neck will lose fast with the increase in the oxide scale (Figure 7c), resulting in the degradation of MSCs. The porosity is another factor to determine the oxidation resistance of metal support [92,93]. Molin et al. [92] found that Fe_2_O_3_ and Cr_2_O_3_ formed on the porous 430 L stainless steel surface, while on the dense 430 L surface, only Cr_2_O_3_ was detected. Then, the sample with Fe_2_O_3_ and Cr_2_O_3_ saw a much higher increased rate (3.3 mΩ cm^2^ h^−1^, in the air at 800 °C for 100 h) in area-specific resistance than that of the sample only with Cr_2_O_3_ (0.25 mΩ cm^2^ h^−1^, in the air at 800 °C for 100 h). This effect is attributed to the high specific surface area of porous structure, which is available for oxidation [92,94].

**Figure 8:**
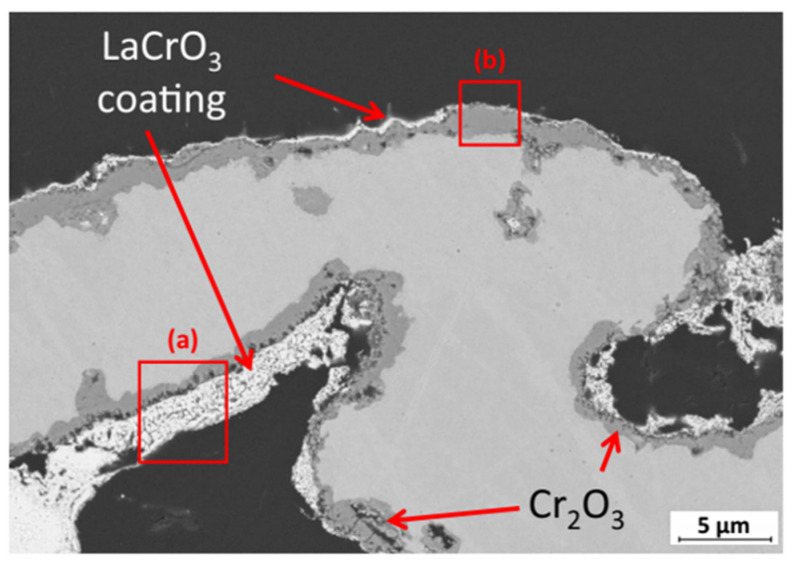
SEM image of porous ITM support after La acetate coating with the. indication of area (**a**) thick and area (**b**) thin LaCrO_3_-coating, reproduced with permission from Ref. [95].

Coating techniques are generally taken into account when it comes to avoiding metal oxidation. However, the inner surface of the porous metal support needs to be coated, which is much more complex than that on a regular flat outer surface. Jeong et al. (2020) [95] coated LaCrO_3_ on the inner surface of the porous metal support (ITM) by the dip-coating method. The microstructure is shown in Figure 8. Although a discontinuous and uneven coating is distributed on the inner surface of the metal support, the oxidation is mitigated. A much smaller oxidation mass gain of 0.2% (at 800 °C in Ar-2.9% H_2_/4% H_2_O over 48 h) with LaCrO_3_ coating was achieved compared with 2.6% of no-coating support. However, significant optimization of the coating is still required for long-time operation and further application. Moreover, to increase the lifetime of metal supports, optimizing structure to avoid small necks and further reducing the operating temperature should also be considered.

#### 2.1.3. Carbon Deposition and Sulfur Poisoning

Hydrogen has been considered an ideal fuel for SOFCs as the product is only water steam and no adverse reaction that damages cells’ performance and lifetime. Compared with hydrogen, carbon-based fuels such as methane and CO can still be an alternative because they are more compatible with existing infrastructures (transportation and storage), reducing industrial application costs. However, when carbon-based fuels are used, the carbon deposition (coking) often happens in the anode, which decreases the performance and lifetime of cells. The following chemical reactions show the mechanism of carbon deposition formation [41]:

(4) $${\mathrm{CH}}_{4}\to C+2H_{2}$$

(5) $$\mathrm{CO}+H_{2}\to C+H_{2}O$$

(6) $$2\mathrm{CO}\to C+{\mathrm{CO}}_{2}$$

**Figure 9:**
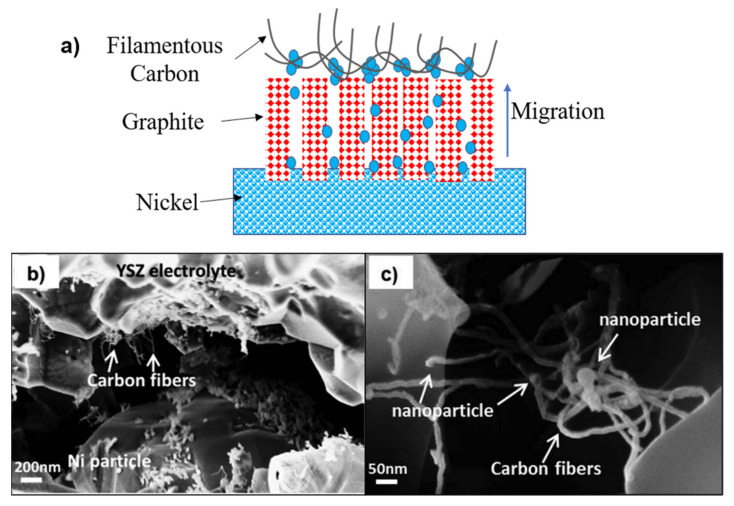
(**a**) Schematic representation of the mechanism of metal dusting corrosion of nickel, adapted from Ref. [43]. (**b**) The interface between the Ni particle (lower part) and the YSZ electrolyte (upper part) close to the gas inlet area of the cell and (**c**) showing the carbon nanofibers and nanoparticles at the Ni-YSZ|YSZ interface, reproduced with permission from Ref. [96].

The precipitation of graphite and other C-based materials on nickel surface through catalytic graphitization mechanism has been long reported [42]. The nickel cations transform to the graphite’s outer surface by graphitic channels and merge to form small nickel particles [4,43,44,45,96]. An example is displayed in Figure 9a. Moreover, these small nickel particles catalyse filamentous carbon (carbon fibres and nanotubes), as shown in Figure 9b and c. The carbon deposition clogs porosity and decreases the catalytic sites’ availability, while the nickel dusting will lead to further degradation.

To mitigate the degradation caused by carbon deposition, some researchers added metal Cu into the Ni-containing cermet anode or replaced Ni with Cu completely since Cu has insufficient catalytic activity for the bond-breaking of C–H and C–C [96,97,98,99,100]. Due to its low catalytic activity, the addition of Cu suppresses the carbon deposition but lowers the power density (only 370 mW/cm^2^ at 700 °C) [97]. In 2020, Li et al. [46] designed a high-performance and high coking resistance MSC with a nickel-manganese spinel anode (Ni-MnO-Mn/Fe-doped GDC). The MSC showed a stable operation at 650 °C in humid H_2_ over 100 h without carbon deposition while a peak power density of 869 mW/cm^2^ was achieved. According to the reference, the high coking resistance was attributed to the adsorption of fine MnO particles on the nickel surface. At the same time, the increased catalytic activity of GDC by doping contributed to the high performance [46]. Although this is the best result to date when CH_4_ is used, the mechanism of the resistance to carbon deposition and enhanced performance is not well clarified, and further research would be needed.

Besides the carbon deposition, the degradation caused by sulfur poisoning also is introduced into the anode when carbon-based fuel is used. H_2_S is often present in methane or CO natural sources. Sulfur poisoning is caused by sulfur adsorption on the nickel surface [101]:

H_2_S (gas) + Ni (solid) → Ni-S (surface) + H_2_ (gas)

**Figure 10:**
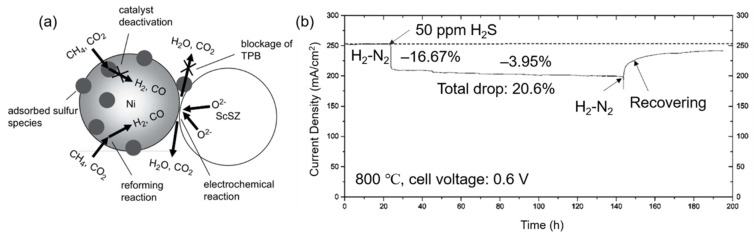
(**a**) Schematic diagram of the degradation mechanism caused by H_2_S poisoning reproduced with permission from Ref. [102]. (**b**) Sulfur poisoning and regeneration or desulphurization processes of Ni-YSZ anodes in a fuel mixture with 50 ppm H_2_S, reproduced with permission from Ref. [101].

Sulfur adsorption mainly leads to two bad effects on cells’ performance, reducing catalytic efficiency by separating nickel with fuel gas and blocking TPBs [102], as shown in Figure 10a. Whether sulfur adsorption or sulfide formation leads to cell degradation was controversial in the early years because the sulfides of nickel were detected in the cell after the operation [49,103]. Cheng et al. (2007) [103] monitored the anode (Ni-YSZ) chemistry during the operation via in situ Raman microspectroscopy. Sulfides were found out to form during the cooling rather than the operation process. Thus, sulfur poisoning is attributed to the sulfur adsorption on the nickel surface rather than the sulfides formation. Moreover, the discovery of the regeneration (or desulphurization) process by oxidation of sulfur species (SO_2_) also gives further support for the sulfur adsorption mechanism [101,104]. Zha et al. (2006) [101] put H_2_S (50 ppm) into fuel gas during the cell operation, saw a decrease of 20.6% in current density, and then stopped H_2_S and found a gradual recovery of performance, as shown in Figure 10b. The recovery is attributed to sulfur desorption from the anode surface by reacting with H_2_ from the fuel and O^2−^ ions from the electrolyte [101,104,105,106]:

(7) $$H_{2}\;\left(\mathrm{gas}\right)+S\;\left(\mathrm{solid}\right)⇋H_{2}\;S\;\left(\mathrm{gas}\right)$$

(8) $$2O^{2-}+S\;\left(\mathrm{solid}\right)⇋{\mathrm{SO}}_{2}\;\left(\mathrm{gas}\right)+4e^{-}$$

The presence of chemical reaction equilibrium is thought to be the reason for failing to complete the performance.

**Figure 11:**
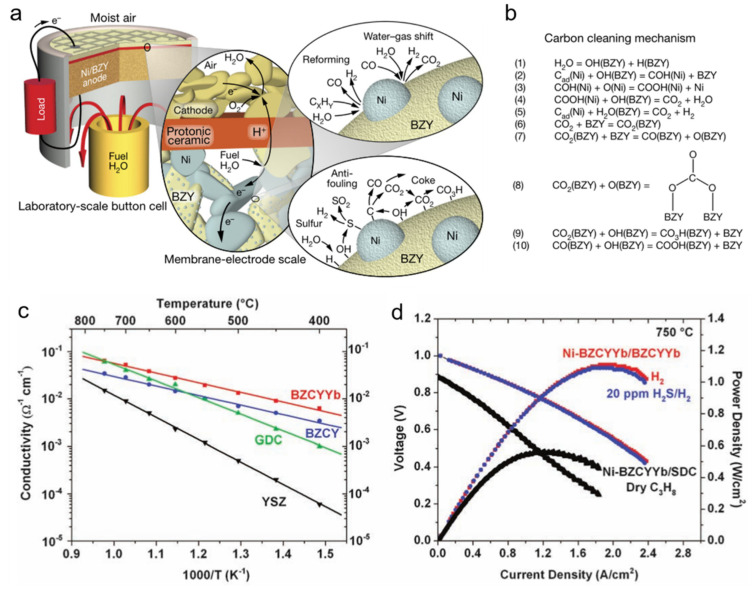
(**a**) Schematic illustration of the protonic ceramic fuel cell (PCFC) and mechanism of hydrocarbon reforming, water-gas shift reaction, sulfur cleaning, and carbon cleaning; (**b**) Mechanism of carbon cleaning. C_ad_ indicates carbon absorbed on the surface of Ni. Reproduced with permission from Ref. [50]. (**c**) Ionic conductivities of BZCYYb, BZCY, GDC, and YSZ measured at 400 to 750 °C in wet oxygen (with ~3 vol % H_2_O). (**d**) Typical current-voltage characteristics and the corresponding power densities measured at 750 °C for a cell with a configuration of Ni-BZCYYb|BZCYYb|BZCY-LSCF when ambient air was used as oxidant and hydrogen as fuel (with or without 20 ppm H_2_S contamination), and for another cell with a configuration of Ni-BZCYYb|SDC|LSCF when dry propane was used as fuel. Reproduced with permission from Ref. [51]. Note BZY: BaZr_0.9_Y_0.1_O_3−δ_; BZCY: Ba(Zr_0.1_Ce_0.7_Y_0.2_)O_3−δ_; BZCYYb: BaZr_0.1_Ce_0.7_Y_0.1_Yb_0.1_O_3−δ_.

Proton ceramics have been found to have high resistance both to carbon deposition and sulfur poisoning due to their high conductivity of protons and oxide ions [50,51]. Mechanisms of carbon and sulfur cleaning were given by Duan et al. (2018) [51] when a Ni-BZY (yttrium-doped barium zirconate, here BaZr_0.9_Y_0.1_O_3−δ_) anode was used, as shown in Figure 11. The ability to adsorb and decompose water of BZY promotes the carbon cleaning reaction. The carbon on the nickel surface will be removed by forming CO_2_ or forming hydrocarbons adsorbed on the surface of BZY last (Figure 11b). Similarly, the formation of OH (BZY) is also regarded as helpful for sulfur cleaning, facilitating sulfur oxidation. In addition to the coking and sulfur poisoning resistance, proton ceramics often shows better conductivity than oxygen-ion conductors at relatively low temperature [50], as shown in Figure 11c. Excellent power density can be achieved when an anode Ni-BZCYYb, an electrolyte BZCYYb, and a cathode BZCY-LSCF were used even in fuel gas containing H_2_S a concentration of 20 ppm at 750 °C (Figure 11d).

Although proton ceramics show excellent applicability for fuel cells (note results above were obtained with anode-supported cells), their use in MSCs remains challenging [107,108]. A summary of the issues (Table 2) was given by Wang et al. (2019) [108]. Although a metal-supported single cell with LCN electrolyte was successfully fabricated by co-sintering, the ohmic cell impedance is too high for actual application [108].

### 2.2. The Degradation of the Cathode

Conventional degradation mechanisms for ESCs or ASCs are commonly listed as following [109]:

(1) Chemical reaction with electrolyte to form insulating phases at interfaces [110,111,112];
(2) Decomposition of the cathode material [5,9,10,62,113];
(3) Delamination of the cathode [114,115];
(4) Coarsening of the microstructure due to sintering [9,109];
(5) Chromium poisoning [53,116].

**Figure 12:**
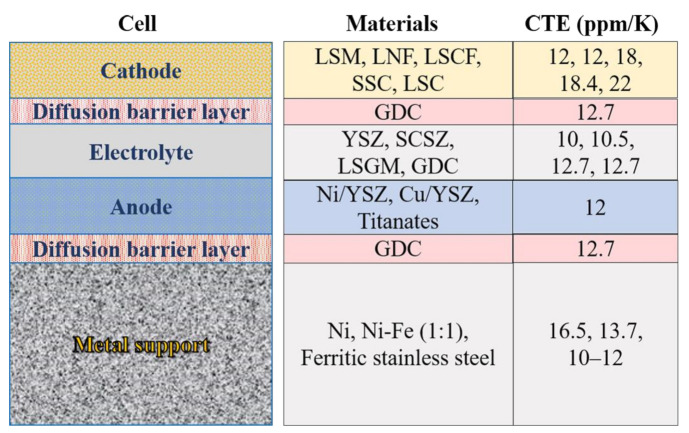
Candidate materials for MSCs, adapted from Ref. [9]. Lanthanum strontium manganite (LSM); Lanthanum nickel ferrite (LNF); Lanthanum strontium cobalt ferrite (LSCF); Strontium samarium cobaltite (SSC); Lanthanum Strontium Cobaltite (LSC); Scandia-ceria-stabilized zirconia (SCSZ); Lanthanum strontium gallium magnesium oxide (LSGM).

Cathode and electrolyte often react at high operating temperatures to generate poorly conductive oxides, resulting in increased resistive phases and performance degradation. Typical phases in zirconia-based SOCs are La_2_Zr_2_O_7_ pyrochlores, caused by the reaction between LaMnO_3_-based cathode and YSZ electrolyte [110,111]. With the decrease in the operating temperature from ESCs to ASCs then to MSCs (Figure 2), the risk of degradation (1) has been dramatically reduced. Moreover, DBL application (Figure 6 and Figure 12) between cathode and electrolyte has further suppressed degradation (1). The co-sintering process mainly causes the decomposition of the cathode material in MSCs. A reducing atmosphere is needed to protect metal support from oxidation during the co-sintering process. In contrast, most cathode catalysts decompose in such an atmosphere [5,9,10]. Therefore, only a few cathode materials that can survive in the reducing atmosphere at relatively low temperatures have been used in MSCs (Figure 12). However, some cathode materials’ CTEs, such as LSCF, LSC, and SSC, are much higher than electrolyte, which increases the risk of cathode delamination [114].

**Figure 13:**
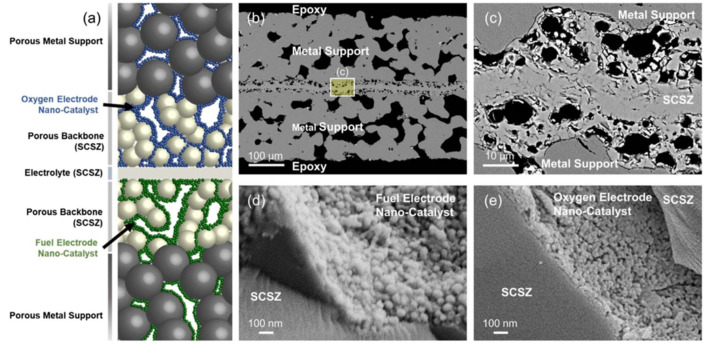
(**a**) Structure diagram of the MSC with infiltrated electrodes. (**b**) SEM micrographs of the cross-section of the MSC showing the “metal-support/SCSZ backbone/SCSZ dense electrolyte/SCSZ backbone/metal-support” symmetric structure. (**c**) Cross-section of porous backbones and dense electrolyte. (**d**) Hydrogen electrode catalyst (Ni-SDC) infiltrated on SCSZ backbone (**e**) Oxygen electrode catalyst (Pr_6_O_11_) infiltrated on SCSZ backbone. Reproduced with permission from Ref. [118].

**Figure 14:**
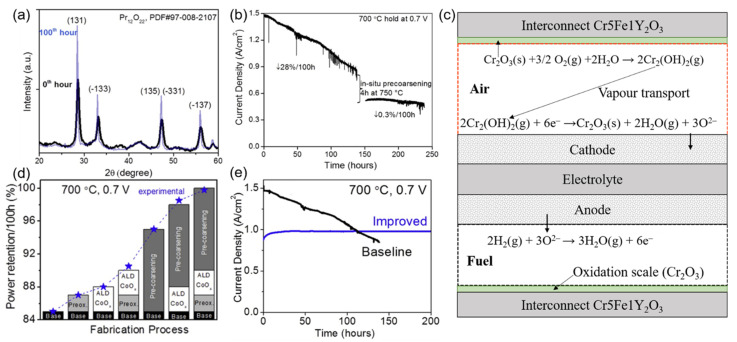
(**a**) Powder X-ray diffraction on PrOx cathode catalyst calcined at 600 °C (black) and after thermal annealing at 700 °C (blue) in the air for 100 h; (**b**) Impact of in situ catalyst pre-coarsening. MS-SOFC durability at 700 °C and 0.7 V before and after in situ catalyst pre-coarsening at 750 °C for 4 h; (**c**) Cr transport at the cathode side of a SOFC; (**d**) Bars represent quantified power retention (percentage of remaining power density after 100 h of operation vs. the beginning of life) observed for the post-sintering fabrication processes, individually and combined. Each process is associated with different colours and marking. Experimental results from whole cells are overlaid (blue stars). (**e**) Long-term durability for baseline and improved cell (after the cathode pre-coarsening, metal support pre-oxide, and ALD process) at 700 °C and 0.7 V. (**a**,**b**,**d**,**e**) were reproduced with permission from Ref. [52]; (**c**) was adapted with permission from Ref. [116].

With the application of infiltrated electrodes, more choices of cathode catalytic materials are allowed because catalytic materials with a large CTE can be infiltrated into the porous cathode backbone in the form of nanoparticles [28,52,117,118]. Then, the mismatching between the cathode and electrolyte can be avoided. Moreover, due to no further requirement of sintering for the infiltrated nanoparticles, the cathodic microstructure’s coarsening caused by the sintering can also be avoided. Wang and Dogdibegovic et al. (2019) [52,118] infiltrated nano-Pr_6_O_11_ into SCSZ (scandia-ceria-stabilized zirconia) backbone to form the cathode (Figure 13), no conventional degradations such as (1), (2), (3), and (4) happened and showed the excellent performance both in SOFC and SOEC mode. However, a new degradation mechanism occurs, which was shown in their successive work [52]. An excessive degradation rate of 28%/100 h (Figure 14b) is mainly caused by the severe coarsening of nano-Pr_6_O_11_ catalytic particles (Figure 14a). Yet, the pre-coarsening method was used as a countermeasure to mitigate the degradation rate. Although a degradation rate of 0.3%/100 h can be achieved after the pre-coarsening process (Figure 14b), the coarsened catalytic particles also significantly reduced the cell’s performance, similar to the technical bottleneck of the early anode (Figure 5a). Pre-coarsening is only a temporary compromise between performance and durability. The solution to this problem is to design stable cathode nanometer catalytic materials. Compared with the infiltration method, ex situ sintering is another method that has been used to suppress the cathode’s degradation in recent years [119,120,121,122,123]. The sintering of the cathode on the whole cell is typically performed in argon at 950 °C. Thus the oxidation of the metal can be avoided while the decomposition of the cathode has been mitigated significantly. However, La_2_O_3_ was found during the sintering when LSC-based cathode was used, which led to the formation of La(OH)_3_ and potentially decreased the performance and durability of cells [121]. LSC/GDC dual-phase cathode was used to suppress the degradation caused by La(OH)_3_ because the rigid GDC network can additional mechanical stability for the cathode, according to Udomsilp et al. (2019) [120].

Besides the mentioned above, chromium poisoning is also one of the main degradation mechanisms of the cathode. Chromium poisoning is mainly caused by the vaporization, migration, and deposition of chromic oxide scales from the cathode-side stainless steel supports or connectors, and the formation of Cr gaseous species can be explained by the following reactions [53,116]:

Cr_2_O_3(s)_ + xO_2(g)_ → 2CrO_i(g)_  (i = 1, 2, 3) (9)

Cr_2_O_3(s)_ + xO_2(g)_ + yH_2_O_(g)_ → 2Cr (OH)_j(g)_  (j = 3, 4, 5, 6) (10)

Cr_2_O_3(s)_ + xO_2(g)_ + yH_2_O_(g)_ → 2CrO(OH)_n(g)_  (n = 1, 2, 3, 4) (11)

Cr_2_O_3(s)_ + xO_2(g)_ + yH_2_O_(g)_ → 2CrO_2_(OH)_k(g)_  (k = 1, 2) (12)

The partial pressure of oxygen and water steam largely determines the formation of Cr gaseous species. The vaporization of Cr species on the SOFC anode could be neglected because the oxygen and water steam pressure are too low around the interface between the anode and metal support (or connector) [53,116]. The process of Cr gaseous species formation and migration is shown in Figure 14c. Cr gaseous species migrate to the electrolyte along with the airflow (the difference in oxygen concentration) and eventually deposit on the cathode surface or the interface between cathode and electrolyte, resulting in the degradation of the cell. Moreover, chromia has been found to react with many cathode materials, leading to the composition change and even the decomposition of cathode materials. Badwal et al. detected (Cr, Mn)_3_O_4_ at the LSM/YSZ interface [124]. E. Konysheva et al. (2006) detected SrCrO_4_ when LSCF (La_0.6_Sr_0.4_Co_0.2_Fe_0.8_O_3_) was used as the cathode [125]. Some researchers suggest mitigating the Cr poisoning by de-humidification or drying of the inlet air [126,127,128,129]. Simultaneously, it is difficult to avoid water vapour in the cell system with the long-term operation. The resistance of different cathode materials to Cr poisoning also has been investigated [130,131,132,133]. LaNi_0.6_Fe_0.4_O_3_ (LNF) was found to have a relatively high tolerance to Cr poisoning since LNF was less reactive with Cr_2_O_3_ compared to LSM (La_0.8_Sr_0.2_MnO_3_) and LSCF (La_0.6_Sr_0.4_Co_0.2_Fe_0.8_O_3_) [130,132]. Cr poisoning indeed can be mitigated by material design and composition optimization. However, the most effective countermeasure is avoiding the formation of Cr gaseous species.

Coating techniques such as atomic layer deposition (ALD) [52,134,135] and atmospheric plasma spraying (APS) [136,137] have been used to fabricate the barrier layer on the Fe-Cr stainless steel surface to avoid direct contact between Cr and moist oxygen. CoO_x_ was deposited on the Fe-Cr stainless steel of air-side in SOFC cathode by ALD from the work of Dogdibegovic et al. in 2019 [52]. The appreciable contribution of the ALD technique to suppress Cr poisoning is shown in Figure 14d. The other two contributions in Figure 14d are the pre-coarsening of nano-Pr_6_O_11_ and the Fe-Cr support’s pre-oxidation. Figure 14e shows the final achievement (“improved” line) after a series of treatments, including pre-coarsening, ALD, and pre-oxidation. Similar to Figure 14b, the degradation rate has been dramatically reduced, but at the same time, pre-coarsening resulted in a significant decline in cell performance.

Overall, the coarsening of nano-catalysis particles is the bottleneck to improve the infiltrated electrode cells’ performance further. Avoiding the coarsening of nanoparticles in electrodes through process optimization and material design should focus on future research.

## 3. The Degradation of Cell Stacks

A cell stack consists of single cells and cell stack components (mainly including interconnects and sealants). Cell stacks’ degradation mechanisms can be concluded as the microstructural degradation of single cells and structural failure of stacks’ components. Single-cell issues have been discussed in Section 2, while structural failure caused by high temperature and thermal stress will be discussed as follows.

Thermal stress during the cell stack operation is mainly caused by the gradient temperature (*G*) from the fuel gas outlet (low temperature) to the inlet (high temperature) [138]. *G* can reach around 200 K in a plane-type ceramic-supported cell stack [139,140], primarily due to the low thermal conductivity of the ceramics. The uneven temperature distribution is easy to generate thermal stress, which leads to the fracture of the brittle ceramic electrolyte or electrodes, resulting in the degradation of the cell stack [140,141,142]. It is widely regarded that the use of metal supports increases the thermal conductivity and the robustness of cell stacks, and the degradation caused by thermal stress has been significantly mitigated [29,30,35]. However, no research on the temperature distribution and thermal stress of MSCs stacks has been reported.

Sealants are essential components of SOCs stacks to avoid gas leakage and mixing. Sealing is a general challenge for SOCs’ durability due to the high temperature and thermal stress during the operation. Compared with the sealing process of conventional SOCs stacks by using brittle glass-ceramic or mica, the metal welding process can be directly used in the sealing of MSCs stacks [9,30]. Uneven-distributed compressive stress to mica can cause significant gas leakage, which leads to the degradation of the cell stack [29], while the welding can avoid this risk. Sudireddy et al. (2017) [30] used the laser welding method to seal the MSCs stack with a degradation rate of 0.5–1.2%/100 h (over 2 kh) at 700 °C in moist hydrogen. A better result by Leah et al. of Ceres Power was reported in 2019, 1 kW stacks with the degradation rate of 0.2%/kh (over 17.6 kh) at 610 °C in reforming gas have been developed, which further promotes the full commercialization of MSCs [143].

Interconnects are essential components for both SOCs and MSCs to connect individual cells, providing conductivity and also mechanical support. Ferritic stainless steels (Cr containing is over 16% normally) are mainly used for interconnects [56,84,93,144]. The degradation related to interconnects is divided into two aspects: one is cathode poisoning caused by Cr evaporation as mentioned in Section 2.2, the other is the conductivity loss caused by oxidation. Thereupon, coating techniques are applied to interconnects to inhibit Cr evaporation and oxidation simultaneously. Table 3 shows the typical materials and coating techniques for interconnects that have been used in recent years.

Spinel and perovskite materials are mainly used as coatings for interconnects. Mn-Co spinel has gained more attention due to its excellent performance and high cost-effectiveness in recent years [144,148,149]. Firstly, Mn-Co spinel coating has better performance both in suppressing Cr evaporation and oxidation than perovskites. Moreover, Mn-Co spinel coating has a high electrical conductivity of over 60 S cm^−1^ at 800 °C, and a closed CTE (9.7 ppm/K) with interconnects (10.5 ppm/K) and is lower cost than perovskites [148,150]. Besides coating materials, coating methods are also significant for the coating quality. In more recent years, high coating density deposition methods such as PVD and APS are regarded as high-quality coating methods for interconnects and have been used mostly [149].

Overall, compared with conventional ceramic-supported cell stacks, the degradation issues caused by thermal stress are mitigated in MSCs stacks because of the use of metals. Subsequently, the oxidation of metals at high temperatures becomes a severe problem for MSCs stacks. Reducing the operating temperature should be the direct way to mitigate the oxidation of metals.

## 4. Thin-Film Electrolyte Metal-Supported SOCs and Issues

Reducing the operating temperature is widely regarded as an effective way to mitigate the oxidation of metal supports. According to oxidation mass gain data of ferritic stainless steel by Molin et al. (2008) [92], a stable *K*_P_ of 0%^2^/h can be achieved at 400 °C in humid H_2_, while the value is 0.029%^2^/h at 800 °C.

**Figure 15:**
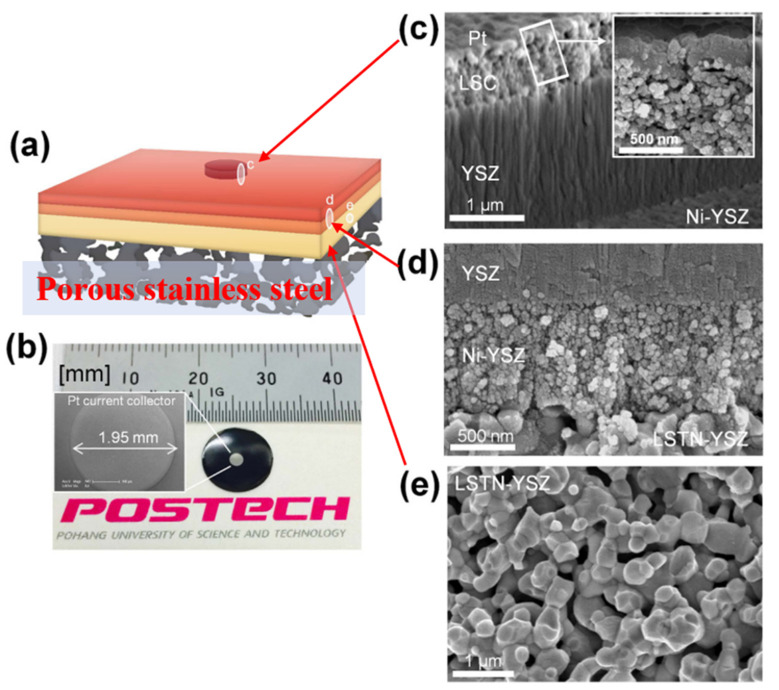
Images of TF-MSC. (**a**) structure diagram; (**b**) morphology and size; (**c**–**e**) Microstructure of the corresponding positions in (**a**). Reproduced with permission from Ref. [19].

The application of advanced technologies such as PLD, ALD, and SPS makes it possible to reduce the thickness of electrolytes to 2 µm and even less than 1 µm [19,20,21,22]. Then, thin-film electrolyte metal-supported SOCs (TF-MSCs) with operating temperatures of below 600 °C have gradually garnered increasing amounts of attention from researchers, especially after 2010 [17,19,20,151]. In 2015, Kim et al. [19] fabricated the TF-MSC with an electrolyte thickness of 2 µm and an active area of about 3 mm^2^ based on the pulsed laser deposition (PLD) method, the structure is shown in Figure 15. The substrate is LSTN-YSZ (40 μm)/porous stainless steel (380 μm) fabricated by tape casting. NiO-YSZ anode, YSZ electrolyte, and LSC cathode were deposited on the substrate in sequence. A peak power density of 560 mW/cm^2^ can be achieved in moist hydrogen at 550 °C while no degradation was observed in both 13-h operation and over 10 thermal cycles. These excellent performances show the promise of this TF-MSC for portable electronic devices that require high power-density and fast thermal cycling. At the same time, the small active area limits further application.

In 2018, Reolon et al. [17] increased the active area of TF-MSC to 38 mm^2^ and further decreased the electrolyte thickness to 890 nm, also based on the PLD method. NiO/ScYSZ anode was deposited on the porous metal support, then YSZ and CGO (GDC) electrolytes with a total thickness of about 890 nm was deposited on the anode successively. Although a peak power density of 400 mW/cm^2^ can be achieved, a significant degradation rate of 10–15%/hour appeared over 20 h test. Degradations are mainly caused by the cracking of the electrolyte and gas leakage. On the one hand, further decreasing the electrolyte thickness will reduce the mechanical strength, resulting in a decrease in thermal stress tolerance [22]. On the other hand, the decrease in the electrolyte thickness will challenge the sealing, increasing the risk of gas leakage [151].

Therefore, while reducing the thickness of the electrolyte, the strength of the electrolyte and the sealing issue should also be taken into account. The balance between the size and the thickness of the film should be concerned. Although the operating temperature has been significantly reduced by using thin-film electrolytes, long-term test data are in shortage.

## 5. R&D Opportunities and Recommendations

**Figure 16:**
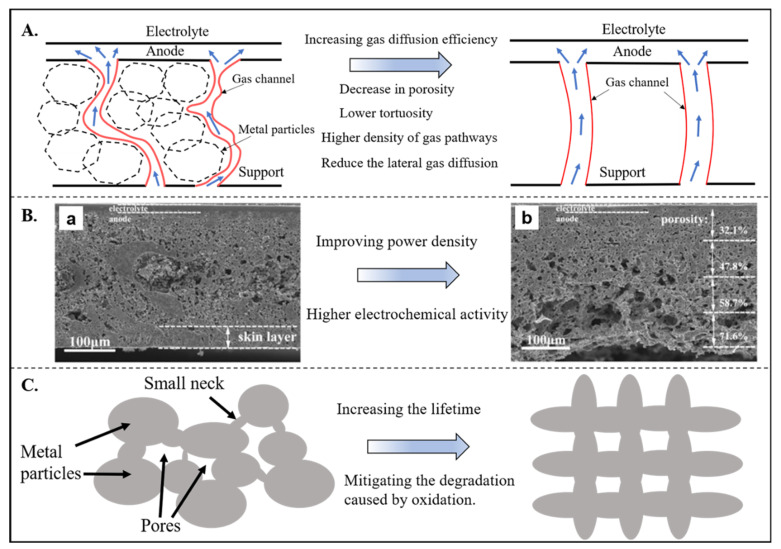
(**A**). Low-tortuosity gas channels have higher gas diffusion efficiency, adapted from Ref. [81]; (**B**). Gradient-porosity anodes facilitate electrochemical reactions, resulting in a higher power density, reproduced with permission from Ref. [152]; (**C**). Regular-shaped and -distributed pores avoid the formation of small necks, increasing the lifetime of MSCs.

Infiltrated electrodes and thin-film electrolytes have been used to improve the performance and durability of MSCs in the past decade. However, the structure of the metal support of MSCs has barely changed in the past two decades. Conventional powder metallurgy methods fabricate typical random-distributed pores and curved gas channels. Such a traditional structure seems to have been unable to meet the needs of high-performance MSCs of the future that requires excellent dynamic performance and long-term stability. Three aspects should be considered to optimize the metal support structure (Figure 16): A. high-efficiency gas diffusion channels; B. gradient-size pores; C. avoiding corrosion-sensitive small necking between metal particles of the support.

Firstly, recent work by Nielsen et al. (2018) [81] achieved more than 40% improvement in power density of the MS-SOFC by using the metal support with straight fuel gas channels, which attributed to the higher gas diffusion efficiency of the straight channels (Figure 16A). The effective diffusion coefficient *D*_eff_ can be represented by the following equation [153]:

(13) $$D_{\mathrm{eff}}=D\;\frac{\varepsilon }{\tau^{2}}$$

where *D* is the bulk diffusion coefficient, *ε* is the porosity, *τ* is the tortuosity. Thus, a larger *D*_eff_ can be obtained by decreasing *τ* and increasing *ε*. Secondly, achieving the gradient-size pores of the anode (including metal support) is also an effective way to improve cell performance [152,154,155]. Smaller pore sizes in the anode’s functional layer help improve the catalytic efficiency as the larger specific surface area can be provided. In comparison, larger pore sizes in the support layer will facilitate gas diffusion. Chen et al. (2014) [152] showed an increase of over 20% in peak power density could be obtained when a gradient-porosity anode is used. Moreover, if small sintering necks (see Section 2.1.2) can be avoided, the degradation of metal support caused by oxidation can be mitigated. Although interconnected porosity can be improved by adjusting parameters including particle size and morphology, sintering temperature, pore former, and organic additives of powder metallurgy [9], the regular-distributed pores and high-efficiency gas channels are difficult to be achieved by powder metallurgy at present. Compared with powder metallurgy, the laser-drilled method is easier to get straight channels to the anode, which has been used in MSCs already [156,157]. However, transversely interconnected pores are difficult to implement by laser drilling because this technique is based on rapidly melting metal foils to form pores, which limits porosity and electrochemical reaction efficiency. Moreover, wet ceramic deposition techniques cannot be used for electrolyte fabrication when the laser-drilled substrate is chosen for support. Additionally, only dry-process such as PLD can be used, which is high-cost and challenging to fabricate large-sized cells [9,158,159]. Metal additive manufacturing has progressed in fabricating porous structure components in recent years [160,161,162,163]. Interconnected pores in three dimensions with apertures of several hundred microns can be achieved by laser or electron beam powder-bed fusion at present, which will be promising to be used in metal support fabrication after the parameters optimization.

Besides the structure optimization of the metal support, proton-conducting electrolytes should be considered for use in MSCs. On the one hand, proton conductors have higher conductivity than oxygen-ion at lower temperatures (400–700 °C); On the other hand, the formation of water steam can be avoided in the support side when a proton conductor is used. Both of which can mitigate the degradation of the metal support.

## 6. Conclusions

Metal-supported oxide cells have come a long way in recent years. Firstly, advances in process and structural design:

(a) The use of infiltrated anode completely avoids the coarsening during the co-sintering.
(b) The application of DBL removes the limitation of the use of ferritic stainless-steel supports, and replacing nickel supports with ferritic stainless steels results in improved oxidation resistance and improved compatibility with ceramics.
(c) The use of thin-film electrolytes reduces the operating temperature to below 600 °C.

Secondly, progress in materials design.

(d) The cermet anodes such as nano Ni/CGO and Ni/SDC are designed to suppress the nano-nickel coarsening, which improves the stability of infiltrated anodes during the operation.
(e) Proton conductors such as BZY, BZCY, and BZCYYb are highly resistant to chromium and sulfur poisoning, which are promising to be used in MSCs.

Although metal-supported oxide cells show excellent performance, especially in rapid startup ability and power density, durability still needs further improvement. The coarsening of the nano-infiltrated cathode structure is a significant factor restricting the stability of the cell. The coarsening mechanism of cathode catalytic nanoparticles and stable cathode catalytic nanoparticles should focus on future research. On the other hand, the structure optimization of the metal supports is expected to improve the cell’s performance and lifetime. To mitigate the degradation caused by metal supports, coating technologies are required for the MSCs fabrication, leading to the high manufacturing cost at the present stage. Thus, to meet the requirement of future high-performance and low-cost MSCs, further technological innovation will be needed both in materials design and process optimization. The work summarized in this paper provides a basis for the direction of innovation.

## Figures and Tables

**Table 1:** The comparison between Ni, Ni-Fe, and 400-series stainless steels [5].

Metal	CTE (ppm/K)	Relative Oxidation Resistance
Ni	16.5	Poor
Ni-Fe (1:1)	13.7	Poor
400-series stainless steel	10–12	Good

CTE of electrolytes (YSZ, CGO, LSGM) are 10–12 ppm/K.

**Table 2:** Summary of proton conductors’ issues in MSCs [108].

Family	Candidate	Representative Composition	Survives Sintering in Reducing Atmosphere?	Survives Re-Oxidation?	React with Metal?	Densifies at 1450 °C or Lower?	Evaporation during Sintering?
Pyrochlore	LCZ	La_1.95_Ca_0.05_Zr_2_O_7_	No	No	-	-	-
	LCO	La_2_Ce_2_O_7_	No	Yes	Yes-Cr, Si	-	-
Perovskite	BCN	Ba_3_Ca_1.18_Nb_1.82_O_9_	Yes	Yes	Yes-Cr, Si	Falls apart	Yes
	BZCY	BaZr_0.7_Ce_0.2_Y_0.1_O_3_	Yes	Yes	Yes-Cr, Si	Marginal	Yes
	SZCY	SrZr_0.5_Ce_0.4_Y_0.1_O_3_	Yes	Yes	Yes-Si	Yes	Yes
Acceptor doped rare-earth Orthoniobate	LCN	La_0.99_Ca_0.01_NbO_4_	Yes	Yes	No	Yes	No

**Table 3:** Coatings of the interconnects.

Interconnects	Coating	Testing Conditions	*K*_P_ (g^2^ cm^−4^ s^−1^)	ASR (mΩ)	Year/Ref.
SUS430	Mn-Co/PVD	800 °C/air/1250 h	1.22 × 10^−14^	28.6	2019/[91]
Crofer22APU	MnCo_1.7_Fe_0.3_O_4_/APS	700 °C/air/1000 h	No data	50	2014/[136]
Crofer22APU	MnCo_1.7_Fe_0.3_O_4_/EPD	800 °C/air/5000 h	0.34 × 10^−14^	No data	2017/[145]
Sanergy HT	(Mn,Co)_3_O_4_/Screen priting	800 °C/air/1500 h	2 × 10^−14^	No data	2011/[146]
AISI 430	Mn-Co/DGPA	800 °C/air	0.25 × 10^−14^ (750 h)	29 (408 h)	2019/[147]

EPD: Electrophoretic Deposition; DGPA: Double Glow Plasma Alloying Process.

## Data Availability

Data sharing is not applicable to this article.
